# Hepatitis B vaccination coverage rates among under-five children in India: a systematic review and meta-analysis protocol

**DOI:** 10.3389/fped.2025.1632476

**Published:** 2025-09-04

**Authors:** K. Divyasree Bhat, Abhinav Sinha, Haimanti Bhattacharya, Yuvaraj Jayaraman, Debdutta Bhattacharya, Sanghamitra Pati

**Affiliations:** ^1^ Department of Microbiology and One Health, ICMR-Regional Medical Research Centre, Bhubaneswar, India; ^2^ The Tamil Nadu Dr. MGR Medical University, Chennai, India; ^3^ Trichy SRM Medical College Hospital and Research Centre, Trichy, India; ^4^ Indian Council of Medical Research, New Delhi, India

**Keywords:** hepatitis B, vaccine coverage, under-five children, India, systematic review, meta-analysis

## Abstract

**Background:**

Hepatitis B virus (HBV) affects an estimated 1.5 million people globally each year, with nearly 296 million living with chronic infection, contributing significantly to cirrhosis, liver cancer, and premature mortality. Despite the availability of a highly effective vaccine, hepatitis B remains endemic in many low- and middle-income countries, including India, due to barriers in healthcare access and low vaccination uptake. While a timely birth dose prevents perinatal transmission, completion of the full vaccine schedule is essential to prevent horizontal transmission—a major mode of infection in India. India introduced the hepatitis B vaccine in select districts in 2002–2003 and scaled it nationally through the Universal Immunization Programme in 2011–2012.

**Objective:**

This systematic review and meta-analysis aims to estimate the pooled coverage of hepatitis B vaccination among children under 5 years of age across different regions of India.

**Methods and analysis:**

A comprehensive literature search will be conducted using PubMed, Embase, and CINAHL databases to identify studies published between 1 January 2000 and 30 May 2024. The search terms will include “Hepatitis B,” “vaccination coverage,” “children,” and “India,” combined using Boolean operators. Two independent reviewers will screen titles, abstracts, and full-text articles for eligibility. The AXIS tool will be used to assess the quality of cross-sectional studies. Pooled coverage rates will be estimated using a random-effects meta-analysis model. Heterogeneity will be assessed using the *I*^2^ statistic and Cochran's *Q* test. Publication bias will be evaluated through funnel plots and Egger's test. Sensitivity and subgroup analyses will be conducted to explore the robustness of results and sources of heterogeneity.

## Introduction

Hepatitis B is a crucial global health concern, particularly in economically underdeveloped and developing countries such as India, where it significantly impacts public health. Hepatitis B virus (HBV) infection can progress to chronic hepatitis, cirrhosis, and liver cancer, making it a crucial target for preventive healthcare measures (1). Among the strategies to combat HBV is vaccination, which has proven to be highly effective. The World Health Organization (WHO) urges the inclusion of the HBV vaccine in national immunization programs, particularly targeting newborns and children under 5 years of age, to prevent both perinatal and horizontal transmission (1).

Despite the availability of effective vaccines, over 27 million children globally are not immunized against the essential doses of vaccinations (2). Every year, approximately 0.1 million out of the 26 million children born in India are at risk of developing chronic hepatitis B (CHB) overall in their lifespan (3). India began its journey toward comprehensive hepatitis B vaccination coverage in 2002–2003, starting with an initial vaccination program in selected cities. This phased approach paved the way for the HBV vaccine inclusion in the nationwide childhood vaccination program by 2011–2012 and supported progress toward the goal of eliminating the disease by 2030 (4). Despite these efforts, achieving uniform and high coverage rates remains a challenge across India’s diverse and densely populated regions.

National and regional averages mask significant diversity in HBV vaccine coverage across India. Of the 640 regions of India, 110 report coverage rates less than 40%. Notably, in 11 districts, primarily in the north region, less than 20% of children have been vaccinated against hepatitis B (5). These figures highlight the urgent need for targeted interventions to improve vaccination rates and ensure equitable healthcare access.

Understanding the current state of HBV coverage of vaccination among under-five children in India is crucial for identifying gaps and strategizing effective public health interventions (6). Despite the nationwide rollout of the hepatitis B vaccine in India, coverage remains uneven across regions. Understanding these disparities is essential for optimizing immunization strategies. This review will provide pooled, region-wise estimates of vaccination coverage. The findings will inform targeted interventions and policy adjustments to improve vaccine uptake.

## Review question

What is the coverage of HBV vaccine uptake among under-five children in India?

## Methods and analysis

This protocol was developed in accordance with the Preferred Reporting Items for Systematic Review and Meta-Analysis Protocols (PRISMA-P) guidelines (7). The systematic review (SR) on hepatitis B vaccination coverage among under-five children in India will be conducted and reported in accordance with the PRISMA guidelines.

## Protocol registration

The study protocol is registered with the International Prospective Register of Systematic Reviews (PROSPERO) under the identifier CRD42024530478 (8). Any amendments to the protocol made during the course of the study will be documented and updated accordingly.

## Criteria screened for eligibility

### Study design

Our systematic review will incorporate observational studies.

### Inclusion

1. Primary research reports (original articles) reporting observational studies including cross-sectional, case–control, and cohort studies
2. Articles reporting hepatitis B vaccination coverage rates in under-five children in India
3. Reports available from National Immunization Programs in the public domain
4. Peer-reviewed articles and gray literature (such as government reports, policy documents, and unpublished theses) that provide sufficient and reliable data on hepatitis B vaccination coverage among under-five children in India

### Exclusion

1. Qualitative studies, editorials, commentary, dissertations, conference proceedings, and reviews
2. *In vitro* and animal studies

### Study population

Prospective SR study targets vaccine coverage among under-five children in India.

### Inclusion

1. Children under the age of 5 years
2. Children vaccinated in India

## Study setting and timeline

This systematic review will encompass all studies carried out in hospital or clinical environments, including specialized hospital setups for HBV. We will include all publications up to the date of the search.

## Search methodology

The basic search strategy will consist of three concepts: hepatitis B, vaccine coverage, and under-five children. The used for hepatitis B include “hepatitis b virus”[MeSH] OR “hepatitis-b”[MeSH] OR “hepatitis-b, chronic”[MeSH] OR “hepatitis-b surface antigens”[MeSH] OR “HBV infection”; for vaccine coverage, “Immunization”[MeSH Terms] OR “Vaccination”[MeSH Terms]; and for under-five children, “Children”[MeSH Terms] AND/OR “Under Five Children” AND/OR “Childhood” AND “India[Title/Abstract]”.

## Updated search strategy

((“Hepatitis B”[MeSH Terms] OR “Hepatitis B virus”[MeSH Terms] OR “HBV infection” OR “hepatitis B” OR “chronic hepatitis B” OR “hepatitis B surface antigen” OR “HBsAg” OR “hepatitis-b”) AND (“Vaccination”[MeSH Terms] OR “Immunization”[MeSH Terms] OR “Vaccination Coverage”[MeSH Terms] OR “vaccine uptake” OR “vaccination rate” OR “immunization status” OR “birth dose” OR “dose completion”) AND (“Child, Preschool”[MeSH Terms] OR “Infant”[MeSH Terms] OR “Newborn”[MeSH Terms] OR “Children under five” OR “Under-five” OR “Childhood” OR “Infants” OR “Newborns” OR “Toddlers”) AND (“India”[MeSH Terms] OR India))

All three concepts will be added using AND, that is, #1 AND #2 AND #3.

## Data extraction (selection and coding)

Two reviewers will independently screen titles and abstracts to identify potentially eligible studies. The full texts of potentially eligible articles will be retrieved and assessed for inclusion by two additional researchers. Any disagreements during this stage will be resolved by a third reviewer with expertise in infectious diseases. A standardized data extraction form has been developed using Microsoft Excel. Data will be extracted from each study on variables such as sample size, sampling method, study design, study setting, sociodemographic characteristics, and hepatitis B vaccination coverage. Two reviewers will independently perform data extraction, and any discrepancies will be discussed and resolved through consensus.

## Risk of bias (quality) assessment

Each included study will be independently evaluated by two reviewers to minimize bias. The risk of bias in observational studies will be evaluated using the Appraisal Tool for Cross-Sectional Studies (AXIS). This tool comprehensively evaluates studies based on 20 questions across five domains: background (1 question), materials and methods (10 questions), outcomes (5 questions), discussion (2 questions), and other characteristics (2 questions). Each domain will be assessed for risk of bias as either “Yes = 1” or “No/Don't Know = 0”. Studies scoring less than 50% will be categorized as highly biased, those scoring 51%–80% as moderately biased, and those scoring 81%–100% as low biased. For case–control and cohort studies, the Newcastle-Ottawa Scale (NOS) will be employed for quality assessment (https://www.ohri.ca/programs/clinical_epidemiology/oxford.asp).

## Strategy for data synthesis

The information will be synthesized qualitatively based on the characteristics of the included studies. In addition, a meta-analysis will be conducted using the “metan” command in STATA 17.0 software (Stata Corp, TX, USA). Heterogeneity will be tested and reported using the *I*^2^ statistic. The estimated effect will be the pooled coverage rate of HB vaccination among under-five children.

## Analysis of subgroups or subsets

The analysis will be stratified by regions of India—east, west, north, south, central, and northeast—as well as by population characteristics, specifically tribal and non-tribal groups.

## Ethical clearance and publication

This systematic review and meta-analysis raises no ethical concerns, as it relies solely on published and non-commercial literature. Since individual patient data are not involved, there are no privacy issues. The findings will be submitted for publication in a peer-reviewed journal and may also be presented at a scientific conference.

## Involvement of patients and community

No individuals were involved at any stage of this study protocol, as it constitutes only the review of existing published data.

## Discussion

This systematic review aims to summarize hepatitis B vaccination coverage among under-five children in India. There is an urgent need for an overall estimation of vaccine coverage of this disease as a step toward elimination. Effective vaccination not only prevents hepatitis B and its severe complications but also reduces the economic burden on the country. Reviewing existing data allows for the evaluation of the effectiveness of current vaccination programs and initiatives. This includes assessing the impact of past interventions and understanding which strategies have been successful or need modification. The evidence generated will support the planning, implementation, and modification of vaccination programs and will be valuable for awareness-building, resource allocation, and policy development. Moreover, the findings will be valuable for raising awareness, informing planning efforts, and guiding resource allocation. Following the protocol's quality standards, this systematic review aims to produce the most robust evidence possible. By compiling information on the clinical features and outcomes of hepatitis B, this review will enrich the currently limited data, offering fresh insights for healthcare professionals and researchers worldwide. The findings of this systematic review and meta-analysis may offer valuable insights for addressing gaps in the timely administration of the birth dose, particularly in rural and underserved areas. It may also inform strategies for improving outreach to under-immunized and high-risk populations, strengthening cold chain and vaccine logistics to minimize missed opportunities, and guiding the design of periodic catch-up campaigns for children who have missed routine doses.
