# Supplementary material for: Hepatitis B vaccination coverage rates among under-five children in India: a systematic review and meta-analysis protocol
Source: Front Pediatr. 2025 Sep 4;13:1632476. doi: 10.3389/fped.2025.1632476 (PMC12443749; doi:10.3389/fped.2025.1632476)
Supplement: Supplementary file 1 [file Table1.docx]

**Supplementary File**

**Table 1: Data extraction sheet**

| **S.l no** | **Study Reference** | **Location/ State** | **Year of study** | **Study design** | **Duration of the study** | **Sample size** | **Male** | **Female** | **Age group**  **(in Months)** | **Sampling Approach**  **(Randon/Non-Random)** | **Study Setting**  **(Community /Facility)** | **Dose wise coverage** | | | | **Overall Reported Coverage (%)** | **Limitations of the study** |
| --- | --- | --- | --- | --- | --- | --- | --- | --- | --- | --- | --- | --- | --- | --- | --- | --- | --- |
|  |  |  |  |  |  |  |  |  |  |  |  | Birth dose Coverage (%) | Penta 1 Coverage (%) | Penta 2 Coverage (%) | Penta 3 Coverage (%) |  |  |
